# The Pandemic in Words: Tracking Fast Semantic Changes via a Large-Scale Word Association Task

**DOI:** 10.1162/opmi_a_00081

**Published:** 2023-06-09

**Authors:** Julieta Laurino, Simon De Deyne, Álvaro Cabana, Laura Kaczer

**Affiliations:** Instituto de Fisiología, Biología Molecular y Neurociencias (IFIBYNE)-CONICET, Buenos Aires, Argentina; Facultad de Ciencias Exactas y Naturales, Universidad de Buenos Aires, Buenos Aires, Argentina; Computational Cognitive Science Lab, Complex Human Data Hub, University of Melbourne, Melbourne, Australia; Instituto de Fundamentos y Métodos y Centro de Investigación Básica en Psicología (CIBPsi), Facultad de Psicología, Universidad de la República, Montevideo, Uruguay; Centro Interdisciplinario en Ciencia de Datos y Aprendizaje Automático (CICADA), Universidad de la República, Montevideo, Uruguay

**Keywords:** diachronic change, word associations, semantic change, COVID-19, semantic similarity, networks

## Abstract

Most words have a variety of senses that can be added, removed, or altered over time. Understanding how they change across different contexts and time periods is crucial for revealing the role of language in social and cultural evolution. In this study we aimed to explore the collective changes in the mental lexicon as a consequence of the COVID-19 pandemic. We performed a large-scale word association experiment in Rioplatense Spanish. The data were obtained in December 2020, and compared with responses previously obtained from the Small World of Words database (SWOW-RP, Cabana et al., 2023). Three different word-association measures detected changes in a word’s mental representation from Precovid to Covid. First, significantly more new associations appeared for a set of pandemic-related words. These new associations can be interpreted as incorporating new senses. For example, the word ‘isolated’ incorporated direct associations with ‘coronavirus’ and ‘quarantine’. Second, when analyzing the distribution of responses, we observed a greater Kullback-Leibler divergence (i.e., relative entropy) between the Precovid and Covid periods for pandemic words. Thus, some words (e.g., ‘protocol’, or ‘virtual’) changed their overall association patterns due to the COVID-19 pandemic. Finally, using semantic similarity analysis, we evaluated the changes between the Precovid and Covid periods for each cue word’s nearest neighbors and the changes in their similarity to certain word senses. We found a larger diachronic difference for pandemic cues where polysemic words like ‘immunity’ or ‘trial’ increased their similarity to sanitary/health words during the Covid period. We propose that this novel methodology can be expanded to other scenarios of fast diachronic semantic changes.

## INTRODUCTION

Language is not set in stone; it is constantly evolving to accommodate new ideas, technologies, and social change. Throughout our lives we are exposed to situations that require a change in the meaning of the words we already know. For example, we have recently learned that ‘cloud’ also makes reference to data storage over the internet, and a ‘bug’ could refer to an error in a computer script. These new meanings did not replace earlier ones but extended the range of applications for these words (Wilkins, 1996). This process is evident in the case of polysemic words: for example, the word ‘run’ has up to 35 different related senses [e.g., “the athlete runs the race,” “the politician runs for election,” or “the car runs on petrol”; Parks et al. (1998); Rodd (2020)]. It is possible to incorporate new meanings and modify the emotional value of certain words according to an individual’s life experiences (Gentsch et al., 2018). Therefore, memory for words is malleable and different aspects of a word’s meaning are subject to being updated or modified (Laurino et al., 2022). In the present study, we are interested in addressing the plasticity of lexico-semantic representations in a large-scale and ecologically valid experiment in the context of the COVID-19 pandemic.

Regarding collective language change, in diachronic (or historical) linguistics, semantic change refers to any change in the meaning(s) of a word over time (Hamilton et al., 2016). Most words have a variety of senses, which can be added, removed, or altered over time while the form remains constant. For example, the word ‘gay’ shifted from meaning “cheerful” or “frolicsome” to referring to homosexuality, and the word ‘awful’ underwent a process of pejoration, as it shifted from meaning “full of awe” to meaning “terrible or appalling” (Simpson et al., 1997). Like any linguistic change, a semantic change is usually slow, not acquired simultaneously by all members of a speech community, and is difficult to predict (Wilkins, 1996). However, since 2020 we have been experiencing the COVID-19 pandemic, one of the most influential global events in modern human history, which also implies a great deal of linguistic change in a short period of time. In just a few months, some already existing low frequency words became commonplace (e.g., ‘quarantine’, ‘immunity’) and others such as ‘bubble’ have acquired new senses (“a social bubble”). Thus, the pandemic offers a unique opportunity to quantify the drift of word concepts (see Carrillo et al., 2015) that could eventually lead to a semantic change. Thus, in this study, we aimed to explore the collective changes in the mental lexicon as a consequence of the COVID-19 pandemic. However, how do we best capture these changes in word meanings in a large group of people? Several studies have derived word embeddings (such as LSA or word2vec) through known historical changes (e.g., Garg et al., 2018; Hamilton et al., 2016; Stella et al., 2020). However, one of the main limitations of natural language-based approaches to meaning is that they only capture a subset of semantic relations that humans know (Thompson et al., 2020). Several studies have shown that they capture the experiential structure of the word only partially. Psychological measurements of meaning can complement purely text-based approaches as they tend to capture experiential, multimodal aspects of semantic representations. For example, a comparison of text-based models with human word associations showed that the latter capture both perceptual (visual) and affective properties that are only partly encoded in language (De Deyne et al., 2020). This paper will investigate whether word associations from the multilingual Small World of Words can provide new insight into diachronic semantic changes.

To investigate semantic change, we will consider the meaning of words embedded in semantic graphs. This follows the seminal work of Collins and Quillian (1969), which represents semantic memory as a network, or graph, in which nodes represent words, concepts or specific characteristics connected to each other. From this perspective, the meaning of a word can be understood as long as its relationship with the other words in the semantic network is studied. In semantic networks, two concepts are semantically related if they are together or close in the network. In this study, semantic networks are obtained from the data derived through word association tasks. These are apparently simple experiments: a word (i.e., the cue) is presented and the subject has to respond with the first word that comes to mind [i.e., the response; see Nelson et al. (2000)]. Despite their simplicity, they constitute a powerful methodological tool to study the internal representations and the processes involved in the meaning of words (De Deyne & Storms, 2008a) since that associations are free from the basic demands of natural language communication (Steyvers et al., 2006). Relative to other tasks, the word association technique provides us with a more general and unbiased approach to measure meaning (Deese, 1965; Mollin, 2009) and provides access to common sense and experiential meaning (Borge-Holthoefer & Arenas, 2009; De Deyne et al., 2021; Liu et al., 2022). This means that a variety of stimuli can be used as cues, regardless of their part-of-speech or how abstract or concrete they are. Taken together, these properties make word associations a useful tool to study words’ internal representations and to determine the plasticity of the lexical-semantic system. In this sense, the “Small World of Words” project (SWOW, De Deyne et al., 2019) is a large-scale collaborative study that has produced sizable norms of free association in languages such as English, Dutch, Mandarin and Iberian Spanish. This database was also recently generated for the Rioplatense Spanish variant, containing more than 13,000 words, collected from more than 60,000 participants in Argentina and Uruguay (Cabana et al., 2023), where we focused our study.

Until now, semantic change has mostly been addressed using word embeddings in large diachronic text corpora against long-term historical changes (e.g., Hamilton et al., 2016). Although powerful, we suggest that these methods lack the psychological foundation available for word associations which take into account individual and demographic information directly (e.g., De Deyne et al., 2017). In this study our aim is to characterize the use of word associations as a valuable tool for understanding the fast evolution of a word’s meaning. We focused on the changes caused by the COVID-19 pandemic, using a large-scale word association task that already had data prior to the pandemic, Small World of Words, SWOW-RP (Cabana et al., 2023). First, we analyzed if certain pandemic-related words (e.g., ‘virus’, ‘vaccine’, ‘mask’, ‘corona’, ‘contact’) drift from their relatively static associations to a new set of linked words. Second, we asked whether there was an increase in response variability, as certain words might have shifted their prevalent associations, as well as incorporated new senses. Thus, we compared the distribution of responses for each cue between the two periods (Precovid and Covid), using a KL divergence measure. Finally, we captured and analyzed semantic shifts by obtaining network-based similarity estimates using the word association data. For this purpose, we evaluated changes in each cue word’s nearest neighbors and changes in their similarity to certain word senses. The possibility of analyzing natural linguistic changes caused by the environment is useful for determining the plasticity of the lexical-semantic system. We aim to demonstrate this in the context of COVID-19, but propose that this methodology could be extended to address linguistic changes in general in a short time.

## METHODS

### Participants

All participants in this study are part of and included in the Rioplatense Small World of Words data (SWOW-RP, Cabana et al., 2023). The following section summarizes the demographic properties of SWOW-RP participants in general and participants who were presented with the COVID-19 cue set in particular.

Participants were recruited online, using a crowd-sourced approach that relied on social media, e-mail, and university websites. Recruitment was oriented towards Rioplatense Native Speakers. The participants consisted of 61,106 volunteers, of whom 51,043 (84%) identified as female, 9,464 (15%) identified as male, and 599 (1%) responded using the unspecified gender category. The average age was 38 years (SD = 15). Besides gender and age, we also collected information about the native language of the participants. Most people were native Argentinian (51%) or Uruguayan Rioplatense speakers (42%), with Argentinean Cordobés (1%) and Argentinean Nor-oriental-guaraní (1%) speakers as the following largest groups represented in the data. Regarding education, most participants indicated that their highest level was secondary school (41%) or university (48%).

For the first two analyses, we filtered the database to include only the target and control cues and their corresponding responses from each period, resulting in a smaller number of participants. The final participant demographics in these two analyses (after preprocessing, see Cabana et al., 2023 for full details) by time period (Precovid and Covid) is shown in Table 1. As seen from Table 1, the Precovid and Covid cohorts were comparable, except for the regional language variant: There were more speakers from Argentina in the second cohort. We will return to this point in the discussion.

**Table 1.** Participant’s demography after preprocessing steps.

	**Precovid, *N* = 17,263**	**Covid, *N* = 4,181**
**Gender**		
Female	13,834 (80%)	3,287 (79%)
Male	3,322 (19%)	846 (20%)
X	107 (0.6%)	48 (1.1%)
**Age**	37 (14)	40 (13)
**Native language**		
Uruguay Rioplatense	10,894 (63%)	1,003 (24%)
Argentina Rioplatense	6,231 (36%)	3,069 (74%)
Argentina Cordobés	40 (0.2%)	48 (1.2%)
Argentina Nor-oriental-guaraní	17 (<0.1%)	19 (0.5%)
**Education**		
None	9 (<0.1%)	5 (0.1%)
Primary	232 (1.3%)	54 (1.3%)
Secondary	6,285 (36%)	1,318 (32%)
University	9,063 (52%)	2,283 (55%)
Posgraduate	1,674 (9.7%)	521 (12%)

### Materials

#### Precovid group.

For SWOW-RP, stimulus materials (cue words) for the Precovid group were constructed using a snowball sampling method as in De Deyne et al. (2019). The final set of cue words consisted of 13,546 Spanish cues.

#### Covid group.

A subset of cues presented during Precovid were selected as stimuli for a follow-up during the Covid pandemic. Cue words for the Covid time-period consisted of 664 Spanish cues that were either target, control or filler Spanish words. A description of each cue type is given in Table 2. The target cues consisted of pandemic, emotion and routine words and were selected with the criteria of implying an association with the COVID-19 pandemic and being present in the SWOW database. Candidate words were screened by the authors taking into account their frequency in newspapers and conversations in social media since the start of the pandemic. Control and filler cues were selected using the snowball procedure described by Cabana et al. (2023) and by checking that they did not present any evident relationship with the pandemics. Importantly, control words were included for comparison with target cues, constituting a baseline of random semantic drift whereas fillers were included to reduce the priming effects among target cues.

**Table 2.** Cue words presented during the Covid time period.

**Cue type**	***N***	**Description**	**Examples**
control	150	Do not present any evident relationship with the pandemic.	rain, gasoline, dragon, wheel
pandemic	107	Incorporated a new sense or modified its more frequent sense since pandemic.	bubble, alcohol, contact, virus
emotion	119	Related to emotions and that may have been affected during the pandemic.	anxiety, pain, funeral, postpone
routine	108	Related to aspects of the routine that were affected by the confinement.	bar, job, tourism, screen
filler	180	Do not present any evident relationship with the pandemic.	decade, town, start, basketball

As we aimed to ensure that the participants knew all cues well, we selected words relatively common in Rioplatense Spanish (with a word frequency greater than 0.03 per million words, determined with the EsPal Latin American Spanish subtitle tokens (Duchon et al., 2013). The imageability, familiarity and concreteness was balanced across the different groups of cue types (see Appendix, Table 3).

### Procedure

#### General procedure.

The SWOW-RP data collection is identical to Cabana et al. (2023), which follows procedures for similar data in Dutch (SWOW-NL, De Deyne & Storms, 2008b; De Deyne et al., 2013) and English (SWOW-EN, De Deyne et al., 2019). A word association task was collected in a cross-sectional sample at two different time points before the COVID-19 pandemic (Precovid) and during the pandemic (Covid). The Precovid period spanned from December 2013 to March 2020, while the Covid period spanned from December 2020 to April 2022.

Participants were instructed that a word would appear on the screen and they were asked to respond with the first three words that came to mind. If they could not think of any further responses, they could indicate this by pressing the “No More Responses” button. If a word was unknown, they were asked to select the “Unknown Word” button. They were also instructed to respond only to the word displayed on top of the screen (not to their previous responses) and to avoid typing full sentences as responses. Each stimulus appearing on the screen was followed by a form consisting of three text fields, one for the first (R1), second (R2), and third (R3) response. Once a field was completed by pressing ‘enter’ or clicking a button, that entry could no longer be changed.

#### Precovid group.

During the Precovid period, each participant was presented with a list of 14 to 18 stimuli. The stimuli presented were selected randomly from those cues with the fewest responses in the current iteration.

#### Covid group.

During the Covid period, each participant was presented with a list of 20 stimuli based on random selection of 4 target cues (pandemic, emotion and routine cues), 4 control and 12 filler cues for each participant. The small proportion of target cues as opposed to control and filler cues was used to prevent participants from perceiving the aim of the experiment and biasing the data.

### Data Preprocessing

#### Exclusions.

We excluded participants from the dataset if they did not meet the a priori criteria used in SWOW-RP. The exclusion criteria are based on Cabana et al. (2023) and are summarized below. First, we excluded participants where more than 30% of the responses consisted of short sentences (494 or 1% of participants). We excluded participants for whom fewer than 80% of the responses were unique (47 or 0.08% of participants). We also removed participants with fewer than 60% of their responses appearing on a Spanish word list (944 or 2% of the participants). Finally, participants who indicated that they did not know more than 60% of their words were excluded (917 or 2% of participants).

In addition to the participant’s exclusion criteria used in SWOW-RP, we filtered the database to include only the target and control cues from each time period. As our study aims to compare the responses of pandemic-related words between the Precovid and the Covid time periods, we included 60 participants per time period (120 participants per cue) for target and control cues. In the cases where more than 60 responses were available for a cue, we preferentially included data from native speakers, first Argentinean and Uruguayan Rioplatense speakers and then Argentinean Cordobés and Nor-oriental-guaraní speakers. Some target cues were not included in the final dataset because they were not presented during the Precovid time period due to a mistake. These were three words from the *emotion set*, eight *pandemic* and four *routine* target cues. The final dataset consisted of 21,444 participants (17,263 Precovid and 4,181 Covid), 469 cues and 168,840 responses.

#### Normalization.

Consistent with the SWOW-RP data in Cabana et al. (2023), the data were normalized in several steps. We first removed tags, quotes, final punctuation, double spaces and other unintended symbols. We manually checked cues and responses that were misspelled multiple times and corrected them. Next, taking into account the difference in participants’ demography in terms of Native Language between the two time periods (Precovid and Covid), we included a step for consistency purposes during the analysis. As some words appeared as responses in multiple forms corresponding to regional variations (e.g., *pileta* and *piscina*, meaning swimming pool), in such cases, we replaced the Rioplatense Argentinean lexical variants with their Rioplatense Uruguayan counterparts (10 responses).

### Data Analysis

All data was analyzed with R (R Core Team, 2021). We performed three exploratory analyses to detect and characterize diachronic changes in a predefined set of words (pandemic, control, emotion, routine).

We first analyzed the proportion of new responses within the Covid group, in order to determine the appearance of new associations for target cues, so that we can effectively confirm that a diachronic change occurred.

Second, we compared the distribution of responses for each cue between the two time periods to characterize the semantic change. To do that, we calculated the Kullback–Leibler (KL) divergence, a non-symmetric measure of the directed divergence between two probability distributions.

Third, we obtained similarity estimates using the word association data and subsequently determined the cue words’ semantic shifts by using the similarity ratio of each cue and its nearest neighbors in both periods as proposed in Rodriguez et al. (2023). Further details on this analysis are presented in the Results section.

Lastly, and as a first approach to a new measurement, we calculated for both periods the similarity between each cue and paradigm words that represented the core of certain word’s senses (e.g., ‘immunity’ could be related to the resistance to a pathogen or to diplomatic immunity). Thus, we were able to determine the shift in similarity from one sense to another across time.

Regarding statistical analysis, the proportion of new responses for each cue was analyzed fitting a generalized linear model using a binomial family and the logit link. KL divergence data was analyzed fitting a linear model using a normal distribution. The similarity ratio was analyzed fitting a generalized linear model using a gamma family and the inverse link, as the data were continuous, non-negative and positive-skewed. The significance of a factor was determined with an F-Test or a Chi-square Test, as appropriate. Pairwise post-hoc analysis was performed using the *emmeans* package (Lenth, 2022) and applied the Tukey correction to the p-values for comparisons. The significance of the shift in similarities between periods was determined with a permutation test by determining the shift of each cue to 1000 random pairs of paradigm words. These were selected from the similarity matrix and each one had a cosine similarity comparable (i.e., ±0.05) to the similarity between the pairs originally used for each sense.

## RESULTS

### Proportion of New Responses

As a first step to detect whether the proposed methodology could be used to detect language changes, we determined whether we could reliably find new senses associated with known target words. If that was the case, new senses would be represented in our data as new responses to a cue. Thus, we determined the proportion of new responses for each cue. A response was considered as *new* if it presented zero frequency at the Precovid time period and a frequency bigger than zero at the Covid time period.

**Figure 1.**
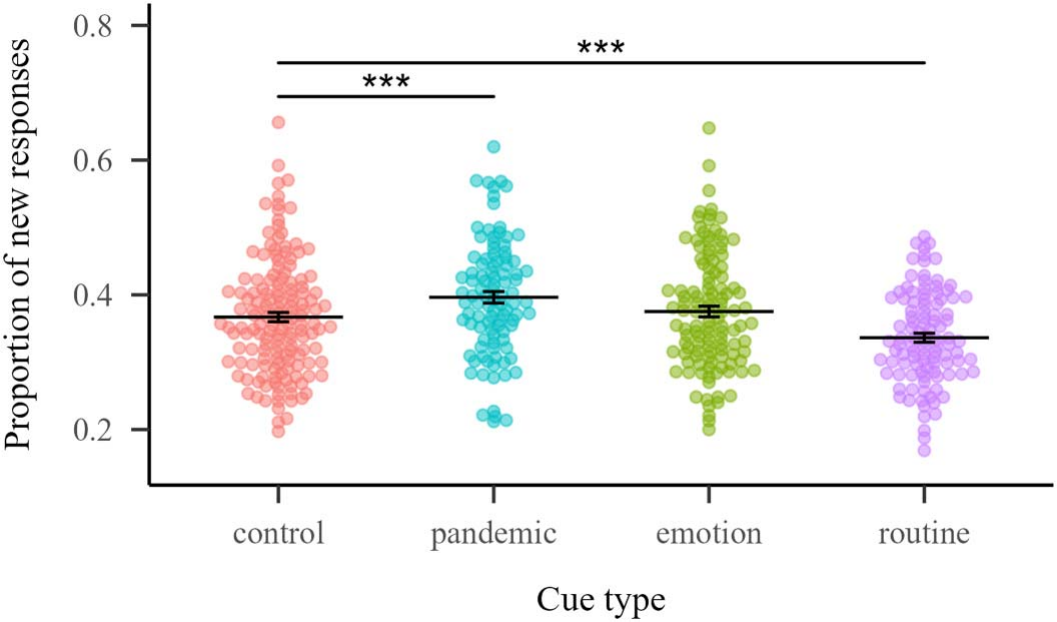
**Proportion of new responses for each cue by cue type.** Horizontal lines indicate the mean of each cue type and error bars the standard error of the mean. Stars indicate statistical differences with the control cue type.

The proportion of new responses by cue was analyzed using a generalized linear model that included Cue type (levels: control, pandemic, emotion and routine) as fixed factor. Statistical analysis revealed a significant effect of Cue type [*χ*^2^(3) = 112.21, *p* < 0.001]. Pairwise simple contrasts revealed that control cues differ significantly from pandemic cues (*p* = < 0.001, Δ = −0.128) and from routine cues (*p* = < 0.001, Δ = 0.130), indicating that pandemic cues presented a greater proportion of new responses than control cues, while routine cues presented a smaller proportion of new responses than control cues (Figure 1).

**Figure 2.**
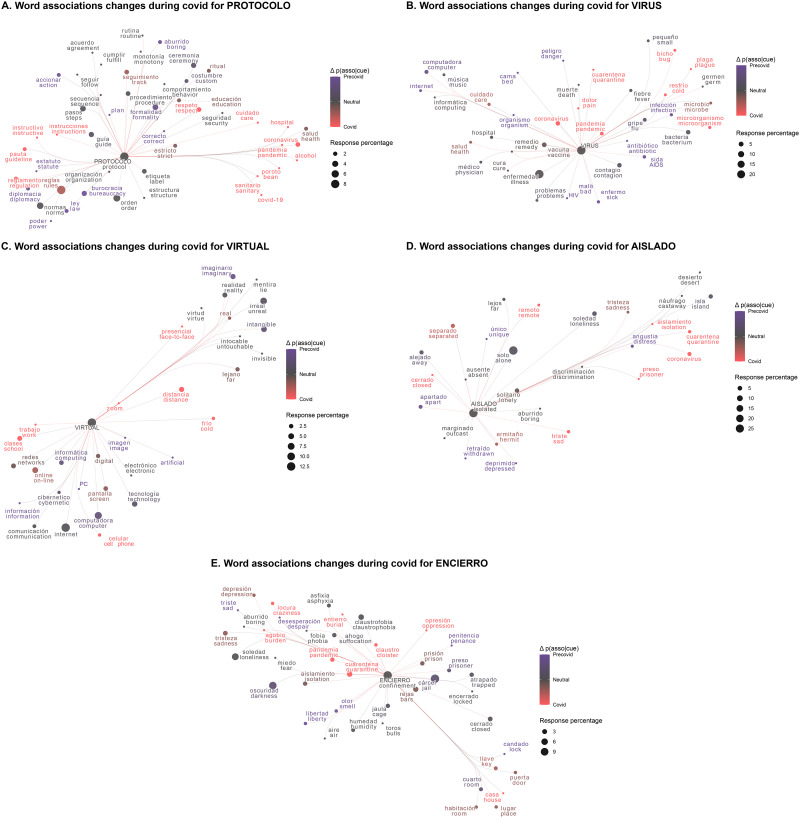
**Network visualization of five pandemic cues with the highest proportion of new responses: ‘protocolo’ (protocol) (A), ‘virus’ (virus) (B), ‘virtual’ (virtual) (C), ‘aislado’ (isolated) (D) and ‘encierro’ (confinement) (E) with their corresponding adjacent vertices.** The node size corresponds to the percentage the response was given for the pandemic cue. The color is based on the difference of the percentage the response was given during the Precovid and during the Covid periods (responses specific to the Precovid period are shown in blue and responses specific to the Covid period are shown in red).

In order to visualize some of these new associations, we selected five pandemic cues with the highest proportion of new responses (see Appendix, Table 4 for a list of the top 10 pandemic cues) and created a semantic network. For each cue, their adjacent vertices (or nodes) were included in the network. To determine adjacent vertices, only outgoing pandemic cue’s edges (or links) were considered, that is, words that were given as a response both during the Precovid and the Covid periods when the pandemic cue was presented. The edges between the adjacent vertices were also included. Words with similar meaning are presented in close proximity by calculating the pairwise cosine similarity and using UMAP (McInnes et al., 2018) to extract a two-dimensional solution that respects both the local and global structure of the similarity space. To reduce visual clutter, an adjacent vertex was included if it occurred at least two times in the entire dataset. Finally, old responses (only given during the Precovid period), new responses (only given during the Covid period) and shared responses (given both in Precovid and Covid periods) were determined and are shown in different colors. As Figure 2D shows, the word ‘aislado’ (isolated) presented new associations during the Covid time period that were tightly related to the pandemic (such as ‘quarantine’, ‘coronavirus’, shown in red). We can observe a similar behavior for the words ‘virtual’, ‘protocol’, ‘confinement’ and ‘virus’ (Figure 2).

### Changes in Response Distribution

In the next section we compare how the meaning of cues changes over time by comparing their response distributions. This would not only consider the new associations but also the changes in response dominance (e.g., it might be possible that during Covid times the cue ‘alcohol’ led mostly to sanitary associations, reducing the connecting strength to drinking associations). To do so, we calculated the Kullback–Leibler (KL) divergence between the Precovid and the Covid probability distributions of responses for each cue.

**Figure 3.**
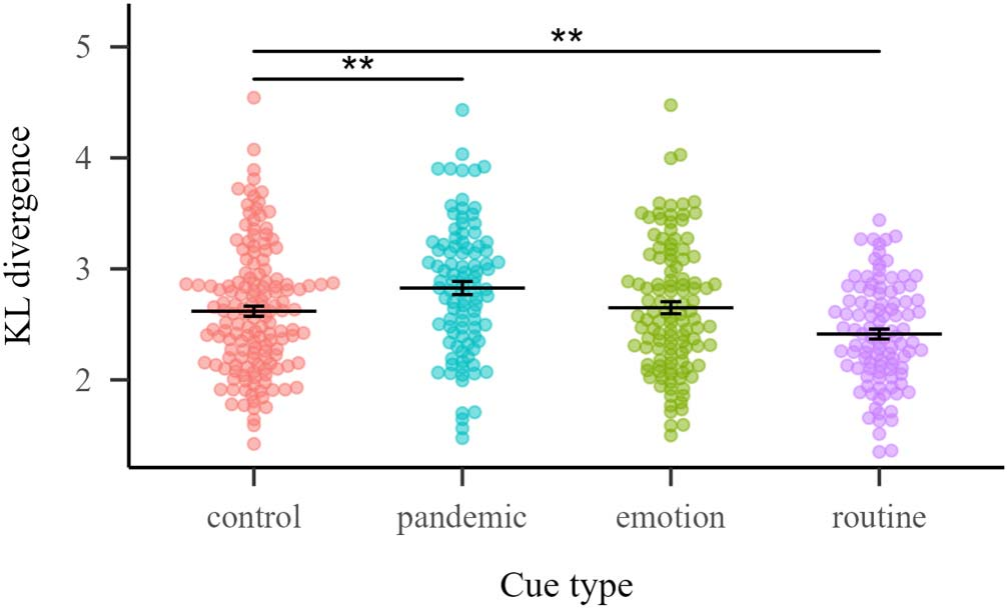
**Kullback–Leibler divergence measure between Precovid and Covid response distributions for each cue, by cue type.** Horizontal lines indicate the mean of each cue type and error bars the standard error of the mean. ** *p* < .01.

We used the *Philentropy* package (Drost, 2018) to compute the KL divergence for each cue in nats (natural units). KL divergence values were analyzed using a generalized linear model that included the fixed factor Cue type (levels: Control, Pandemic, Emotion and Routine). Statistical analysis revealed a significant effect of Cue type [*F*(3, 465) = 9.59, *p* < 0.001]. Pairwise simple contrasts revealed that control cues differ significantly from pandemic cues (*p* = 0.02, Δ = −0.209) and routine cues (*p* = 0.02, Δ = 0.206), indicating that pandemic cues presented a greater KL divergence between Precovid and Covid periods than control cues, while routine cues presented a smaller KL divergence between Precovid and Covid time periods than control cues (Figure 3), suggesting stable meaning compared to controls. See Appendix, Table 5 for a list of the top 10 pandemic cues with highest KL divergence.

### Analyzing Semantic Shifts through Similarity

So far, we determined that pandemic cue words present a greater proportion of new responses and a greater divergence in the distribution of responses between Precovid and Covid time periods. The third measure we present makes use of the fact that word associations can be used to produce good predictions of similarity judgements (De Deyne et al., 2019). Here, we propose that changes in a word’s meaning can be captured by changes in its similarity with other words.

First, we asked whether cue words would present more changes in their most similar words (i.e., nearest neighbors) compared to control words. Second, we explored whether the changes detected reflect a drift to a pandemic/sanitary/health dimension.

As we wanted to measure the similarity between the words during Precovid and during Covid we constructed two different datasets: the Precovid set that only included the responses given in the Precovid period (for the target and control cues); and the Covid set that only included the responses given in the Covid period (for the target and control cues). In contrast to previous analyses, we used the entire SWOW-RP data comprising 56,379 participants, 13,468 cues and 2,508,660 responses. In other words, both datasets shared the responses given for all the cues, except the ones that were target or control. Then, we use these two word association datasets to construct networks that connect associated words and assume that similarity between two words reflects not only the immediate neighbors of a word but also indirect paths, consistent with a spreading activation mechanism (Collins & Loftus, 1975). More precisely, we implemented a spreading activation mechanism as a decaying random walk process (see De Deyne et al., 2019). The procedure is summarized here. We first constructed a cue-by-cue count matrix, where elements reflected how often each cue was given as an association in response to each other cue. Rows of this matrix were normalized to sum to 1 and the positive pointwise mutual information (PPMI) transformation was applied, obtaining a transformed matrix *P*. Then, the distributional representation of each cue was calculated as a matrix *G* by simulating an attenuated random walk over *P* using the equation: *G* = (*I* − *αP*)^−1^, where *I* is the identity matrix, and *α* a decay parameter set to 0.75, the default value used in previous work (e.g., Cabana et al., 2023; De Deyne et al., 2019). Finally, we calculate the similarity between two words as the cosine between rows of *G*. In this context, the similarity between pairs of words is captured by the distributional overlap of the direct and indirect paths they share. This procedure was performed for the two databases separately, obtaining two similarity matrices: one for the Precovid and one for the Covid time periods.

In order to determine whether cue words present more changes in their most similar words (i.e., nearest neighbors) compared to control words, we first determined the top 20 nearest neighbors (i.e., the 20 most similar words) of each cue for each time period. This resulted in each cue having nearest neighbors in the Precovid period, in the Covid period and shared between the two time periods. Then we calculated the ratio of Covid to Precovid similarities between each word and all of its nearest neighbors (cosine similarity ratio). Hence, a ratio of 1 would indicate that the words have the same cosine similarity in both datasets (Precovid and Covid), while departures from 1 would indicate they are more similar at one time period. The cosine similarity ratio was analyzed using a generalized linear mixed model that included Cue type (levels: control, pandemic, emotion and routine) and Neighbor type (levels: precovid, covid, and shared) as fixed factors, and random intercepts for cues. Statistical analysis revealed a significant interaction between Cue type and Neighbor type [*χ*^2^(3) = 90.58, *p* < 0.001]. Pairwise simple contrasts revealed that control cues differ significantly only from pandemic cues and only for the covid Neighbor type (*p* = 0.015, Δ = 0.041), indicating that pandemic cues presented higher similarity ratios for covid neighbors than control cues.

**Figure 4.**
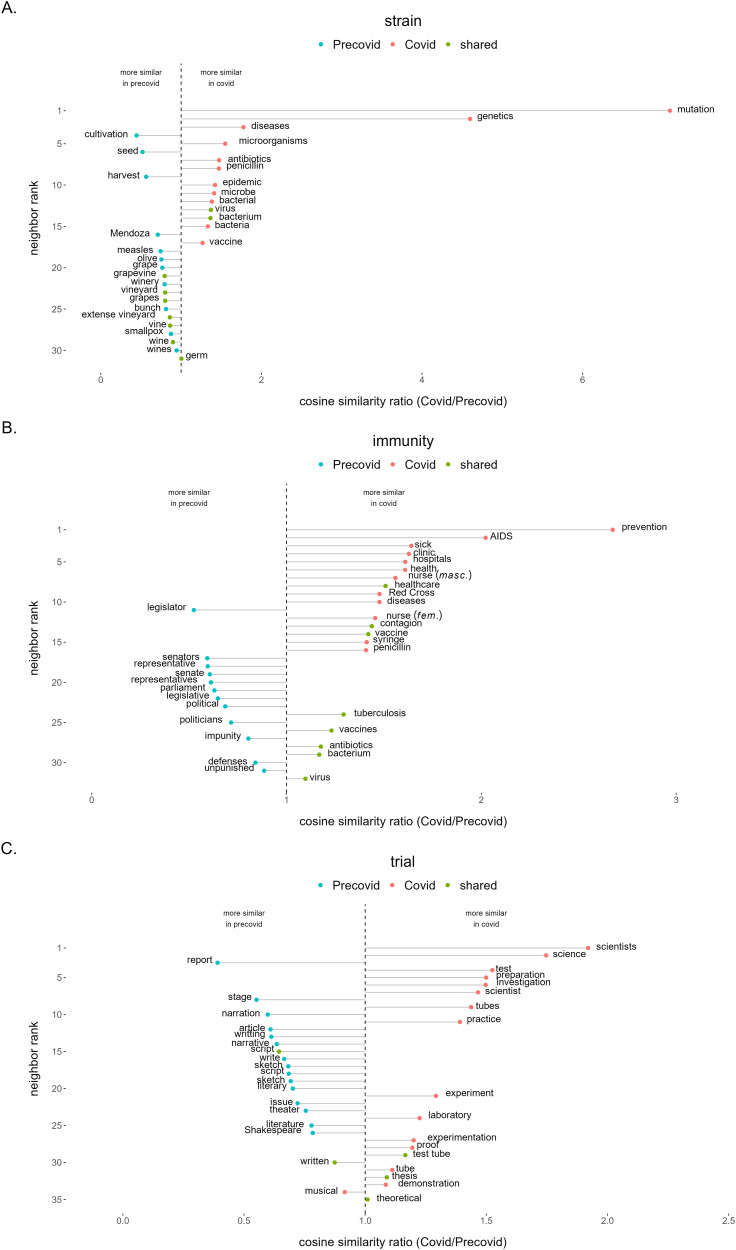
**Ratio of Covid to Precovid similarities between the words ‘strain’ (A), ‘immunity’ (B) and ‘trial’ (C) and their nearest neighbors in the Precovid period (blue) in the Covid period (pink) and shared between the two time periods (green).** When the ratio is large, it means that word is more related to the Covid sense of the cue than to the Precovid one. The y-axis indicates the word’s rank in terms of distance to a ratio of 1 (dashed vertical line) from the most distinct at the top to the less distinct at the bottom.

Consistent with the rationale of this methodology, we show here the results corresponding to three of the cue words with the highest mean ratio deviation from 1 (calculated as the absolute difference between the similarity ratio and 1): ‘strain’ and ‘immunity’ and ‘trial’ (Figure 4). See Appendix, Table 6 for a list of the top 10 pandemic cues with the highest ratio deviation.

In Figure 4A, we observe that the word ‘strain’ presents mostly Covid nearest neighbors concerning infectious diseases (‘genetics’, ‘mutation’, ‘diseases’, ‘microorganisms’, ‘antibiotics’). In contrast, the dominant terms in the Precovid period were related to wine (‘cultivation’, ‘seed’, ‘harvest’, ‘Mendoza’). In Figure 4B for the case of ‘immunity’, we can distinguish a set of specific terms in the Covid period that are health-related (‘prevention’, ‘AIDS’, ‘sick’, ‘nurse’), opposite to Precovid where most terms relate to politics (‘legislator’, ‘senators’, ‘senate’, ‘representative’). Finally, in Figure 4C, the word ‘trial’ presents mostly Covid nearest neighbors concerning experimental and scientific trials (‘science’, ‘investigation’, ‘tubes’, ‘laboratory’). In contrast, the dominant terms of ‘trial’ (also meaning ‘essay’ in Spanish) during Precovid were related to literature and theater (‘stage’, ‘narration’, ‘writing’, ‘script’).

**Figure 5.**
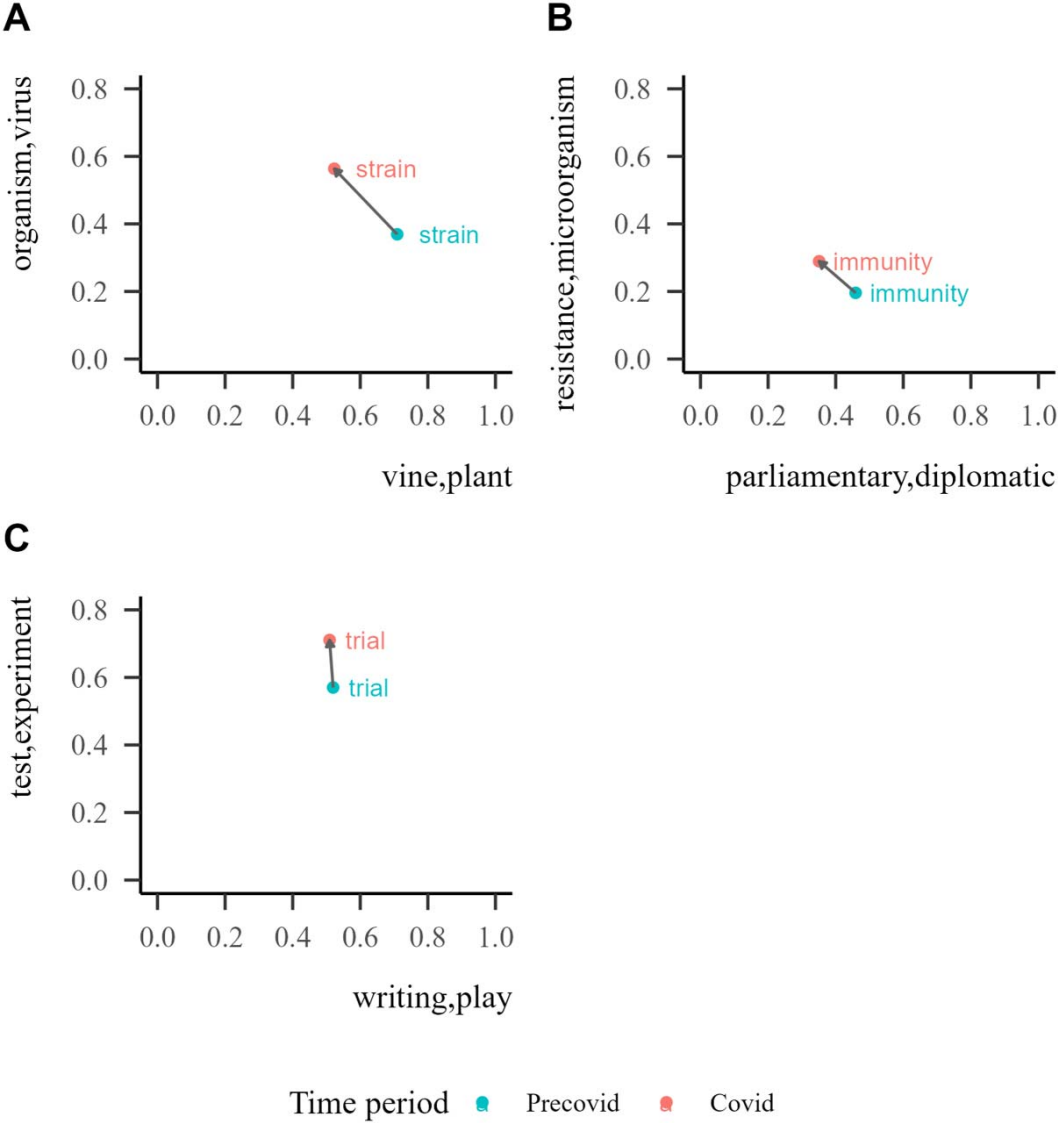
Semantic similarity ratings to each sense for the pandemic cues ‘strain’ (A), ‘immunity’ (B) and ‘trial’ (C), for the Precovid and Covid time period.

Lastly, as a first step in the development of a new measurement of semantic change, we explored whether the changes observed in the nearest neighbors for the three examples reflect a shift to a pandemic/sanitary/health dimension. To do this, we used the information revealed by Figure 4 and calculated the similarity of each word (‘strain’, ‘immunity’ and ‘trial’) to specific word senses (cf. Grand et al., 2022, for a similar procedure), shown in Figure 5. For each cue, we selected two paradigm words that would represent the anchor of two different senses: the dominant sense in the Precovid period and the sense that we predicted increased its dominance during the Covid period. The two pairs of paradigm words were taken from the definitions found in the dictionary of the Real Academia Española (RAE) (2001) and in the WordReference Spanish Dictionary (Landesman, 2002). A cue’s final rating for each of the senses was the sum of the word’s similarity towards each sense’s paradigm words. The two ratings were determined for the Precovid and the Covid period. We conducted a permutation test to establish the significance of the shifts by calculating the shift of each cue to randomly chosen pairs of paradigm words. We observe that, for the three words explored, the shift in the predicted direction is statistically significantly larger than its permuted value, *p* < 0.001. This result indicates that, over many possible directions, semantic drifts move the cues closer to pandemic-related senses.

## DISCUSSION

Understanding how words change their meanings over time is relevant to studies of language and cultural evolution. Semantic change is usually tracked through the collection and careful inspection of data that span decades or centuries (e.g., Boleda, 2020; Garg et al., 2018; Hamilton et al., 2016). However, the COVID-19 crisis has influenced our daily discourse drastically in a short period of time. The present study aims at developing a novel method to address this rapid semantic change, specifically the shifts in lexical meaning in the recent history of Rioplatense Spanish. By using a free word association task we were able to capture subtle and informative changes in lexical mental representations, comparing it with Precovid data. We found that several words drifted from their previous semantic neighborhoods, now revealing associations to the COVID-19 pandemic. In addition, we developed several measurements such as the proportion of new responses, the divergence of response distributions, and measurements of semantic shifts that could be used in other scenarios of diachronic semantic change. This method might therefore be useful to understand fast changes in meaning in a collective scenario.

Three different word association measures were found to detect changes in a word’s mental representation from Precovid to Covid times. First, we found that significantly more new associations appeared for a set of pandemic-related words. These new associations can be interpreted as the incorporation of new connotations and senses. In addition, the introduction of new associations reduces the probability of generating word associations related to Precovid times. To visualize and interpret these changes in individual words we performed a network analysis for the words with the highest number of new associations (e.g., ‘isolated’, ‘protocol’, ‘virtual’, ‘confinement’ and ‘virus’, see Figure 2 and Table 4). Visual inspection of the graphs supports changes consistent with our lived experience of the pandemic. For example, the word ‘isolated’ incorporated direct associations with coronavirus and the pandemic. It is important to consider that many words have several alternative meanings (i.e., homonyms and polysemous words) that might have changed their frequency of usage during the Covid times. In this sense, our methodology could be useful to measure these dominance changes through time. For example, for the cue word ‘virtual’ several associations such as ‘imaginary’, ‘unreal’ and ‘intangible’ are lost, while associations related to technological virtuality appear (e.g., ‘school’, ‘work’ and ‘Zoom’), thus reinforcing the idea of a rearrangement in the semantic network during Covid period. This is remarkable, considering that participants are asked to give three responses, so they do get an opportunity to give responses related to the old senses, but our results show that either old or new senses would prevail.

Second, when analyzing the distribution of responses, we observe a greater KL divergence for pandemic words. This may be because pandemic-related words changed their meanings (increasing their ambiguity or number of senses) due to the COVID-19 pandemic, and thus presented a higher relative entropy of the distribution of responses in Covid time with respect to Precovid time. We have also found that the ‘routine’ words show a reduction in their proportion of new responses and KL divergence with respect to control words. We speculate that this effect might be related to a reduced use of routine words (e.g., related to transportation, leisure, clothing) during the Covid period. In this sense, it has been proposed that less frequently used words would be less prone to semantic drift (Bybee, 2006, but see Hamilton et al., 2016). That is, these words would be less used and therefore their plasticity would be downgraded, leading to a reduced change in response patterns.

Finally, using a semantic similarity analysis we found a greater difference between neighbors in the Precovid and Covid periods for pandemic cues, compared with control cues. In this sense, polysemic words like ‘immunity’ or ‘trial’ were found to increase their sanitary/health association during the Covid period. In this case, word’s senses were determined by manual inspection of the nearest neighbors in each time period and by using this information to select paradigm words from dictionary entries. However, further work to automatically determine each word’s sense might be useful in order to scale this analysis to all words and, therefore, be able to develop a new measure of semantic drift.

We consider that this might be a first stage in a semantic broadening process, by which the meaning of a word evolves over time to represent a more general concept or thing than it did originally (Hamilton et al., 2016). The described methodology could be used in a variety of different scenarios to analyze semantic changes in language usage over time, like tracking changes in politically-related words (Rodriguez et al., 2023), in scientific terminology (e.g., related to climate change or emerging technologies), or cultural shifts. By analyzing the nearest neighbors and tracking changes in semantic similarity over time, we could gain insights into the underlying factors driving changes in language usage.

### Limitations

One limitation of the present study is that it only involves two time periods, not allowing us to measure the persistence of the observed changes. We aim to perform a follow-up study to determine whether the shift in some associations still persists after the COVID pandemic impact has passed. We predict that for some words (such as ‘virus’ or ‘virtual’) the changes were so dramatic that surely we will still find these new associations through several years to come.

An additional caveat of the present study is that the comparison between Precovid and Covid times was performed in a different set of participants. Although it would be interesting to have a within-participant comparison to address individual changes, we consider that given the sample size our study deals with collective language properties that are representative of the population under study (Wertsch & Roediger, 2008). In addition, there was a difference in the regional variant of native Rioplatense Spanish between the Precovid and Covid datasets, where the former included more participants of the Uruguayan variant while the latter had more participants that selected the Argentinean variant. Although this might have led to differences unrelated to COVID, we consider it is unlikely that regional differences account for the results observed for several reasons. First, Uruguayan Spanish and Argentinian Spanish are two linguistic variants of Rioplatense Spanish that share many distinctive phonological, grammatical and lexical features (Di Tullio & Kailuweit, 2011). These substantial lexical characteristics justify the construction of unified lexical norms specific to the Rioplatense dialect, as was done in the SWOW-RP norms (Cabana et al., 2023). Second, none of the most common new responses given during the Covid period (Figure 2) were lexical variants or homophones of Uruguayan and Argentinian Spanish. This was verified by authors native to each of the two countries. Third, we controlled for the regional variations that we detected as responses (even though they were not the most common new responses for target words) by replacing the Argentinean Rioplatense lexical variants with their Uruguayan Rioplatense counterparts (10 responses). Finally, it is important to consider that country-specific patterns during the pandemic could have differentially affected the observed lexical-semantic changes. While both Uruguay and Argentina experienced large degrees of mobility restrictions in 2020 (Cabana et al., 2021; Fiori et al., 2022) that negatively impacted several aspects of everyday life, the governmental response was different in both countries. Argentina underwent a severe lockdown while in Uruguay schools were closed and social gatherings were restricted primarily during the first half of 2020 and 2021, and the resulting mobility reduction had an important voluntary component (Cabana et al., 2021). In any case, the potential interaction effects between nationality and the pandemic would not invalidate our claims that we are indeed measuring semantic shifts that can be attributed to the COVID-19 pandemic and its major social and behavioral changes. Furthermore, considering that the pandemic had a global impact, it would be interesting to expand the measures to other languages as well. In this sense, the Small World of Words project currently contains large datasets in English, Dutch, Iberic Spanish, Mandarin, Cantonese, German, Italian and Japanese, making it likely to address the dynamics of various languages.

### Relevance

We consider that the present study is relevant as a methodological approach to semantic change in an ecologically valid setting. In this sense, one advantage of the current word association task is that it was not framed within a pandemic-related context, but instead it was the context that imposed itself.In other words, we did not lead participants to find any connection with the pandemic, because the target words were presented in isolation and ‘diluted’ within fillers.

Word associations can usefully complement text-based analysis by providing insight into conceptual changes through a controlled procedure in which words are presented relatively context-free, which, consistent with previous work, taps into multimodal and common-sense representations (De Deyne et al., 2021; Liu et al., 2022; Simmons et al., 2008). In addition, while distributional semantics based on text corpus has tremendous potential to accelerate research in semantic change (Boleda, 2020), a major challenge is that these methods need very large datasets, which are usually available in English. Moreover, the vast majority of studies focus on the Google Books Ngrams corpus, which covers only the period between 1850 and 2009 (Boleda, 2020). Word associations thus offer a viable alternative to identify meaning changes in a demographic-aware fashion, for underrepresented languages for which large diachronic corpora are not available.

### Conclusion

This study introduces a new research paradigm for investigating semantic change in the context of COVID-19, which utilizes a large-scale word-association task in two different times. This approach combines methods from computational linguistics (such as semantic networks, and natural language processing techniques) and psycholinguistics (demographic-aware experiment design with selected stimuli). Importantly, the data was obtained without any contextual reference, just using isolated words. Thus, the pandemic context imposed itself, making it one of the rare cases of natural experiments being employed in linguistics. Since research questions of this nature are not usually not tested on an individual’s scale but instead rely on large linguistic corpora, we suggest that the development and adaptation of this research methodology is an important contribution of this study.

Furthermore, we demonstrated that the semantic associations collected for several selected words changed after the COVID-19 pandemic, compared with associations collected before the pandemic. Although this is an intuitive result, it nevertheless shows that semantic associations in aggregated groups of real people are measurably influenced and undergo change in a short period of time, a noteworthy result.

## ACKNOWLEDGMENTS

We thank Nicole Coaker for help with the initial data acquisition and Dr. Luz Bavassi for helpful suggestions during data analysis.

## DATA AVAILABILITY STATEMENT

The dataset presented here is available for research purposes only under the Creative Commons Attribution-Noncommercial-NoDerivs 3.0 Unported License. No distribution or repackaging is allowed without explicit consent from the last author of this paper. Please refer to this dataset as SWOW-RP-COVID word association data.

Raw and processed data are available at https://smallworldofwords.org/uy/project/research. Scripts to process are available through a GitHub repository linked on the same page.

## APPENDIX

**Table 3.** Mean frequency, imageability, familiarity, and concreteness for each cue type.

**Cue type**	**Frequency**	**Imageability**	**Familiarity**	**Concreteness**
control	11.7	5.1	5.6	4.9
pandemic	9.8	4.6	5.6	4.5
emotion	13.6	4.8	5.7	4.5
routine	16.4	5.7	6.2	5.3

*Note*. Frequency, imageability, familiarity, and concreteness for each word was determined with the EsPal Latin American Spanish subtitle tokens.

**Table 4.** Top 10 pandemic cues with highest proportion of new responses.

**Cue**	**Prop. of new responses**
protocolo [protocol]	0.62
responsabilidad [responsibility]	0.57
medidas [measures]	0.57
virus [virus]	0.57
restricción [restriction]	0.56
virtual [virtual]	0.56
normalidad [normal]	0.55
aglomeración [agglomeration]	0.54
aislado [isolated]	0.5
encierro [confinement]	0.5

**Table 5.** Top 10 pandemic cues with highest KL divergence.

**Cue**	**KL**
protocolo [protocol]	4.43
virus [virus]	4.03
responsabilidad [responsibility]	3.92
virtual [virtual]	3.9
medidas [measures]	3.9
restricción [restriction]	3.89
normalidad [normal]	3.89
aglomeración [agglomeration]	3.62
aislado [isolated]	3.57
social [social]	3.55

**Table 6.** Top 10 pandemic cues with highest mean similarity ratio deviation from one.

**Cue**	**Ratio deviation**
cepa [strain]	0.6
inmunidad [immunity]	0.44
mutación [mutation]	0.43
metros [meters]	0.38
garganta [throat]	0.34
fase [phase]	0.33
ensayo [trial]	0.33
grupo [group]	0.32
esperanza [hope]	0.32
adultos [adults]	0.31
